# Enhancement of electroporation facilitated immunogene therapy via T-reg depletion

**DOI:** 10.1038/cgt.2014.35

**Published:** 2014-07-18

**Authors:** P F Forde, M Sadadcharam, L J Hall, T R O' Donovan, M de Kruijf, W L Byrne, G C O' Sullivan, D M Soden

**Affiliations:** 1 Cork Cancer Research Centre, Leslie C. Quick Jnr. Laboratory, BioSciences Institute, University College Cork, Cork, Ireland; 2 Norwich Medical School, University of East Anglia, Norwich Research Park, Norwich, UK

## Abstract

Regulatory T cells (T-regs) can negatively impact tumor antigen-specific immune responses after infiltration into tumor tissue. However, depletion of T-regs can facilitate enhanced anti-tumor responses, thus augmenting the potential for immunotherapies. Here we focus on treating a highly aggressive form of cancer using a murine melanoma model with a poor prognosis. We utilize a combination of T-reg depletion and immunotherapy plasmid DNA delivered into the B16F10 melanoma tumor model via electroporation. Plasmids encoding murine granulocyte macrophage colony-stimulating factor and human B71 were transfected with electroporation into the tumor and transient elimination of T-regs was achieved with CD25-depleting antibodies (PC61). The combinational treatment effectively depleted T-regs compared to the untreated tumor and significantly reduced lung metastases. The combination treatment was not effective in increasing the survival, but only effective in suppression of metastases. These results indicate the potential for combining T-reg depletion with immunotherapy-based gene electrotransfer to decrease systemic metastasis and potentially enhance survival.

## Introduction

The incidence of one of the most intractable forms of skin cancer, malignant melanoma, continues to rise, while at the same time current treatment regimes show limited impact on survival.^1^ Existing treatment strategies include surgery, chemotherapy and radiotherapy, however many patients succumb to local regional and distal reoccurrences.^2, 3, 4^ Current management of melanoma relies on primary prevention and early detection of disease^2, 5^ and new treatment approaches that are more tolerable, reduce the risk of relapse and do not impinge on the patient's quality of life are urgently required.

Conventional therapies have demonstrated poor anti-cancer effects for reasons such as chemoresistance and rapid metastasis.^6, 7, 8, 9^ Due to its inherent poor prognosis compounded by ineffective treatment regimens, there is a significant drive to design diverse treatment strategies against melanoma, with immunotherapy representing a key therapy focus.^10^ The goal of immunotherapy is to increase overall anti-tumor immunity and thus represents a potent means for cancer treatment. However, a major obstacle to the success of immunotherapy is the presence of negative factors that inhibit the immune system such as regulatory T cells (T-regs).^11^ T-regs have been implicated as one of the major suppressive mechanisms of anti-tumor immune responses. Increased levels of T-regs in tumors are often associated with poor clinical outcome and tumor progression in various tumor entities.^11^ In healthy immune homeostasis, T-regs (a subset of T cells) play a crucial role in maintaining immunological unresponsiveness to self-antigens and in suppressing excessive immune responses that would otherwise be harmful to the host.^12^ However, in the tumor environment, T-reg-induced immune suppression poses a significant barrier to anti-cancer responses targeted by immune-therapeutic strategies.^12, 13, 14^ We, and other groups, have demonstrated that an increase or decrease in T-regs has a direct influence on the effect of an immunotherapy approach.^15^ Other studies have used different therapeutic approaches including interleukin-12 and interleukin-2, which have had significant therapeutic responses.^16, 17, 18^ We have previously reported that an immunotherapy DNA plasmid encoding the cytokine granulocyte macrophage colony-stimulating factor (GM-CSF; pGmCSF-b7.1), combined with pre-conditioned T-reg depletion, was effective in the treatment of murine fibrosarcoma.^19^

To determine the efficacy of immunotherapy in aggressive malignant melanoma we electrically delivered a plasmid encoding GM-CSF, in combination with T-reg depleting anti-CD25 to mice receiving the melanoma tumor cell line B16F10. Our results demonstrate that this combinational approach significantly reduced tumor volume and systemic lung metastases and significantly improved overall survival time, and thus represents a promising therapeutic approach against melanoma.

## Materials and methods

### Animals and tumor induction

Female C57BL/6 (6–8 weeks) mice were obtained from Harlan Laboratories (Oxfordshire, UK). All *in vivo* experiments were approved by the ethics committee of University College Cork and carried out under licenses issued by the Department of Health, Ireland, as directed by the Cruelty to Animals Act Ireland and EU Statutory Instructions. Mice were kept at a constant room temperature (22 °C) with a natural day/night light cycle in a conventional animal colony. Standard laboratory food and water were provided *ad libitum*. The B16F10 cell line was obtained from the American Type Culture Collection and cultured (Manassas, VA, USA) in Dulbecco's modified Eagle's medium supplemented with 10%, v/v, fetal calf serum, 300 μg ml^−1^ L-glutamine. Tumor cells from a mid-log phase culture were collected by brief exposure to 0.05% trypsing/EDTA solution, washed twice with phosphate-buffered saline. For routine tumor induction, 5 × 10^5^ tumor cells suspended in 200 μl of serum-free Dulbecco's modified Eagle's medium were injected subcutaneously into the flank of mice. Following tumor establishment, tumors were allowed to grow and develop and were monitored by alternate day measurements in two dimensions using vernier calipers. Tumor volume was calculated according to the formula *V*=*ab*^2^/6, where *a* is the longest diameter of the tumor and *b* is the longest diameter perpendicular to diameter *a*. From these volumes, tumor growth curves were constructed. Mice were culled when the tumor diameter was approximately 1.5 cm.

### Plasmids

The mammalian expression vector pMG was purchased from Invivogen (Cayla SAS, Toulouise, France). A version of this plasmid, designated pGT141, containing the murine GM-CSF and human B71 genes transcriptionally controlled from the hEF1–HTLV and CMV promoters, respectively, was designed and cloning was performed on contract by Invivogen. Human B71 cDNA has been shown previously to function in a murine setting. The inserts were confirmed by sequencing. Plasmids were propagated in *Escherichia coli* strain Top10 and purified on endotoxin-free Qiagen-tip 500 columns (Qiagen, Manchester, UK).

### *In vivo* T-reg cell depletion

The anti-CD25 monoclonal antibody (mAb; clone PC61) was used to deplete and deactivate T-regs in mice. Mice were treated with anti-CD25 mAb by intraperitoneal injection in 0.25 ml endonucleas-free phosphate-buffered saline 1 day prior to performing experiments and every 4 days post tumor induction. Rat IgG1 (HRPN, anti-peroxidase horseradish) was used as a control (Bio-Express, West Lebanon, NH, USA). In previous studies the minimum dose required for each mAb was 1 mg kg^−1^ delivered intraperitonealy to achieve >95% reduction in systemic T-reg numbers without nonspecific T-cell activation.^15^ Validation that the control Ab had no effect on T-reg numbers was achieved using fluorescence-activated cell sorter.

### Flow cytometry analysis

Fluorescence-activated cell sorter analysis was performed using anti-mouse PE-foxp3, FITC-CD4 and APC-CD25 and relevant isotype controls as per manufacturer (eBiosciences, Insight Biotechologies, London, UK). Single-cell suspensions from the spleens and tumors of individual mice were prepared to obtain a final concentration of 5 × 10^5^ cells per well in blocking buffer (1 × phosphate-buffered saline/1% bovine serum albumin/0.05% sodium azide/1% rat, hamster and mouse serum). A quantity of 50 μl of each mAb dye and 5 μl of the amine-reactive viability dye ViViD (Invitrogen) to determine dead cells was added to the blocking buffer. This was then incubated in the dark at 4 °C for 30 min. Cells were washed twice with blocking buffer and finally resuspended in 200 μl 1% paraformaldehyde. To perform flow cytometric analyses and measure relative fluorescence intensities a FACS-Aria cytometer and BD Diva software (BD Biosciences) were used. For each mouse, 20 000–200 000 events were recorded. The percentage of cells labeled with each mAb was calculated in comparison with cells stained with isotype control Ab. Background staining was controlled by labeled isotype controls (eBiosciences) and fluorescence-minus-one controls. The results represent the percentage of positively stained cells in the total cell population exceeding the background staining signal. For determination of intracellular foxp3 production, cells were then fixed and saponin permeabilized (Perm/fix solution, eBiosciences) and incubated with mAb or isotypic control mAb. After 30 min, cells were twice washed in permeabilization buffer (eBiosciences) and then analyzed by flow cytometry as described above.

### *In vivo* electrogenetherapy

When tumors reached approximately 100 mm^3^ in volume (5–7 mm major diameter), mice were randomly divided into experimental groups and subjected to specific experimental protocols. The procedure was carried out under general anesthesia, by intraperitoneal administration of 200 μg xylazine and 2 mg ketamine. The skin overlying the flank tumor was shaved. Fifty μg of plasmid DNA in 50 μl sterile injectable phosphate-buffered saline was injected into the tumor. Two-needle array electrodes (Cork Cancer Research Centre, Cork, Ireland) were inserted on either side of the marked DNA injection point 80 s after DNA delivery for electroporation. The distance between the electrodes was 4 mm. *In vivo* electroporation parameters were 1200 V cm^−1^ 100 μs pulse length; 1 pulse and 120 V cm^−1^ 20 ms; 8 pulses at 1 Hz, and were administered in sequence using the *E.Pore Gx* (Cork Cancer Research Centre, Cork, Ireland) square wave generator. The high-voltage pulse was used to induce electroporation in the cell membrane and the ensuing small voltage pulses were used to create an electrophoretic field to assist movement of the negative charged DNA plasmid across the cells.

### Statistical analysis

Experimental results were plotted and analyzed for significance with Prism 4 software (GraphPad software, La Jolla, CA, USA). *P*<0.05 was considered significant.

## Results

### Suppression of primary tumor growth following administration of anti-CD25 mAb

**Figure 1:**
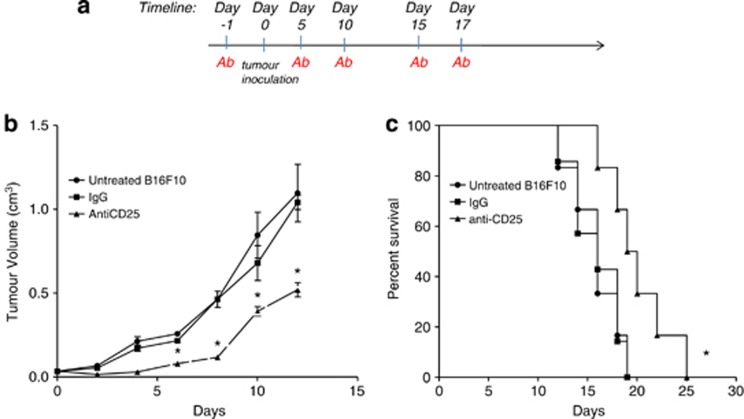
Tumor growth for anti-CD25 monoclonal antibody (mAb) treatments. (**a**) Experimental protocol. C57/BJ6 mice (*n*=6) were treated, that is, with anti-CD25 mAb, irrelevant IgG mAb or untreated 1 day prior to tumor inoculation and 4 days post tumor inoculation, 5 × 10^5^ B16F10 cells were inoculated subcutaneously to the mice and tumour volume was monitored. (**b**) Tumor growth curves. Tumor growth from anti-CD25 mAb, irrelevant IgG mAb and untreated is presented as tumor volume measurements from day 0 to day 12. Tumor volume was calculated using the formula *V*=*ab*^2^/6. Data are presented as the means±standard error of the mean. *At days 6, 8, 10 and 12, compared anti-CD25 mAb with untreated and IgG mAb, *P*<0.05. (**c**) Survival of tumor-bearing mice. Survival is presented using Kaplan–Meier survival curves. *Compared anti-CD25 mAb with untreated B16F10 and IgG mAb, *P*<0.02.

Mice were injected intraperitonealy with anti-CD25 mAb (PC61) or rat IgG1 1 day prior to subcutaneous injection of B16F10 cells and every 4 days post tumor inoculation (Figure 1a).^20^ Subsequent monitoring of growth demonstrated that tumors grew significantly more slowly (*P*<0.05) in the presence of anti-CD25 mAb when compared to untreated mice that had received no antibody or isotype control (immunoglobulin G, IgG; Figure 1b). Irrelevant rat IgG mAb when administered at an equivalent dose and time had no significant effect on growth or survival when compared with untreated tumors. Survival was improved significantly (*P*<0.02) when compared to the untreated group and the irrelevant antibody-treated group from days 19 to 24 (Figure 1c).

### Immunogene therapy suppresses the tumorigenicity of murine melanoma B16F10 cells

**Figure 2:**
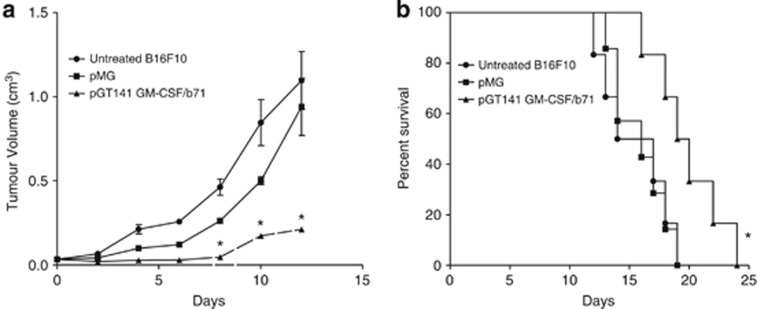
Treatment of preestablished B16F10 using immunogene therapy. (**a**) Tumor growth of pGT141 GM-CSF-b7.1, pMG and untreated of preestablished B16F10 tumor is presented from day 0 to day 12. Data are presented as the means±standard error of the mean. *At days 4, 6, 8, 10 and 12, compared pGmCSF-b7.1 with untreated B16F10 tumor, *P*<0.05. (**b**) Survival of immuogene-treated tumor-bearing mice. Survival is presented using Kaplan–Meier survival curves. *Compared pGmCSF-b7.1 with untreated B16F10 tumor, *P*<0.02.

B16F10 mouse melanoma is considered a poorly immunogenic tumor (American Type Culture Collection). C57BL/6 mice were challenged with 5 × 10^5^ B16F10 cells subcutaneously and subsequently electroporated with pGmCSF-b7.1, pMG or untreated when tumors reached approximately 100 mm^3^ in volume. Notably, the immunogene group treated with pGmCSF-b7.1 showed significant reduction in tumor growth when compared with the untreated group (*P*<0.05), data presented in Figure 2a. This reduced tumor volume also correlated with significantly improved survival (*P*<0.02), however the immunogene therapy was not curative (Figure 2b).

### Combinational therapy of anti-CD25 and immunogene of B16F10 primary tumor

**Figure 3:**
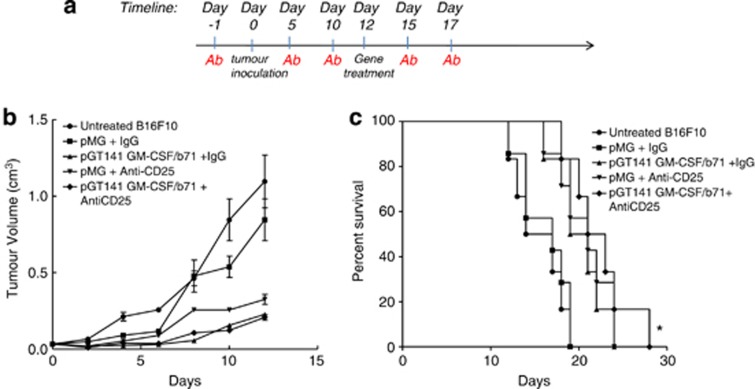
Combination treatments with anti-CD25 and immunogene therapy. (**a**) Protocol of antibody administration, immunogene therapy and tumor inoculation. C57/BJ6 mice (*n*=6) were treated using the antibody regime previously described and combined with immunogene when tumor reached approximately 100 mm^3^ in volume, 5 × 10^5^ B16F10 cells were inoculated subcutaneously to the mice and tumor was monitored. (**b**) Tumor volume was measured and presented as means±standard error of the mean. *Combinational treatment (pGmCSF-b7.1 and anti-CD25 mAb) compared with untreated B16F10, *P*<0.005. (**c**) Survival is presented using Kaplan–Meier survival curves. *Compared pGmCSF-b7.1/anti-CD25 mAb with untreated B16F10 tumor, *P*<0.02. mAb, monoclonal antibody.

To determine how a combinational therapy approach might affect tumor growth, we examined mice treated with a combination of immunogene therapy and anti-CD25 mAb. Mice received either anti-CD25 or rat IgG1, B16F10 cells and were then electroporated with pGmCSF-b7.1, pMG or untreated when tumors reached approximately 100 mm^3^ in volume (Figure 3a). The pGmCSF-b7.1 and anti-CD25 mAb combination treatment regime significantly reduced the growth of the tumor when compared with the untreated B16F10 tumor (*P*<0.05). There was no difference in growth rate when pGmCSF-b7.1 and anti-CD25 combination group was compared with pGmCSF-b7.1 and IgG group and pMG and anti-CD25 group (Figure 3b). Importantly, those mice receiving pGmCSF-b7.1 and anti-CD25 have significantly improved survival (*P*<0.05) compared to the untreated group (Figure 3c). Overall, the combinational therapy approach had similar primary tumor outcomes when compared to either modality alone. There was no improvement in the combination over the single and modality treatments.

### Suppression of pulmonary metastasis from B16F10 tumor

**Figure 4:**
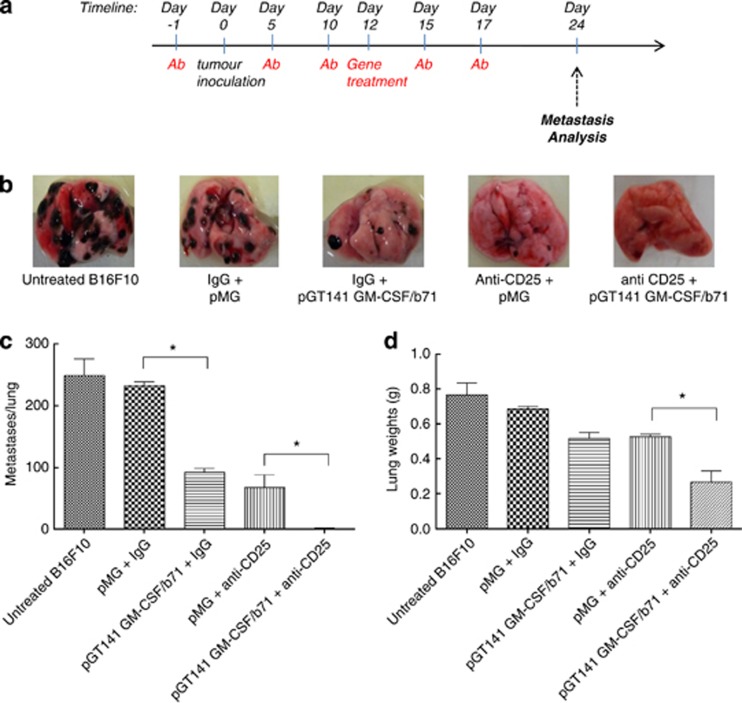
Suppression of pulmonary metastasis from B16F10 tumor. (**a**) Schematic representation of treatment schedule: C57/BJ6 mice (*n*=6) were treated, that is, with anti-CD25 mAb, irrelevant IgG mAb or untreated 1 day prior to tumor inoculation and 4 days post tumor inoculation, 5 × 10^5^ B16F10 cells were inoculated subcutaneously to the mice and tumor volume was monitored. Tumors were treated with±pGmCSF-b7.1 or pMG when the tumors reached approximately 100 mm^3^ in volume. (**b**) Macroscopic image of lungs. (**c**) The number of surface metastases counted. The results represent those from four animals per group. (**d**) Lung weight measurements are presented as means±standard error of the mean. *pGmCSF-b7.1 and anti-CD25 mAb combinational treatment compared with pMG and anti-CD25, *P*<0.05.

We next sought to examine how effective the pGmCSF-b7.1 and anti-CD25 combination would be against metastatic disease. Twenty-four days post primary tumor induction (Figure 4a), lungs were collected, weighed and photographed to determine the number of metastatic nodules on the lung surface (Figure 4b). In untreated mice with a primary B16F10 tumor, we observed approximately 165 metastatic nodules on the lung surface (Figure 4c). Mice in the single therapy groups (that is, pGmCSF-b7.1 and IgG control or control pMG and anti-CD25) had significantly less metastatic nodules when compared with untreated or pMG and IgG combination (*P*<0.05). Notably, only one mouse (out of six) receiving the combination therapy (pGmCSF-b7.1 and anti-CD25) displayed any metastatic lung nodules and thus this group had significantly reduced (*P*<0.05) lung metastases when compared to single therapy or control groups.

### T cell changes during treatment

**Figure 5:**
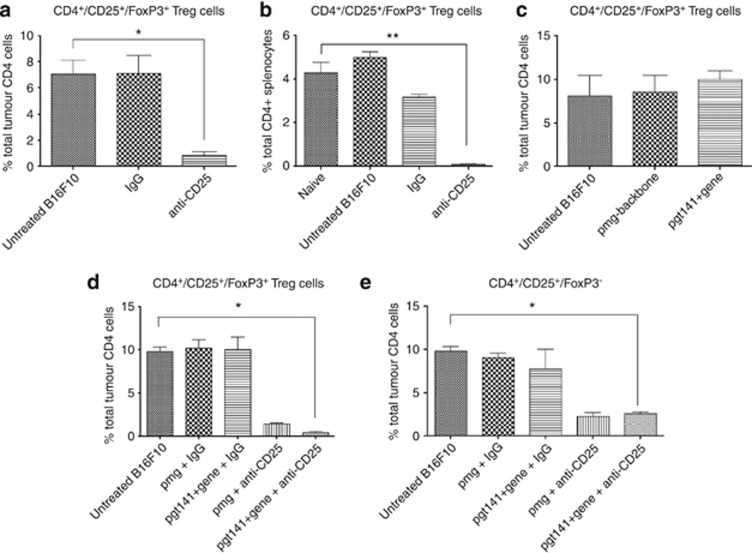
T-regs inactivation. Anti-CD25 mAb intraperitoneal administration inactivates T-regs locally and systemically at the tumor site and spleen. (**a**) The CD4^+^CD25^+^FoxP3^+^ cell populations were significantly reduced. Compared anti-CD25 mAb with untreated B16F10 and IgG mAb in tumor, *P*<0.004. (**b**) The CD4^+^CD25^+^FoxP3^+^ cell populations were significantly reduced. Compared anti-CD25 mAb with untreated B16F10 and IgG mAb in the spleen, **P<0.001. (**c**) The immunotherapy with pGmCSF-b7.1 alone had no effect on the CD4^+^CD25^+^FoxP3 tumor cell populations. (**d**) Combinational treatment reduced the CD4^+^CD25^+^FoxP3^+^ cell population. (**e**) Combinational treatment also reduced the CD4^+^CD25^+^FoxP3^−^ cell population compared with the untreated tumor, *P<0.05. Data are presented as % respective cell population in the respective tissue±standard error of the mean.

In order to assess the effect the therapy had on T cells systemically and locally in the B16F10 tumor model, we collected tumor and spleens from anti-CD25 mAb, pGmCSF-b7.1 and the combination of pGmCSF-b7.1 and anti-CD25 mAb treatment groups. As expected, the anti-CD25 mAb-treatment group significantly depleted the CD4^+^CD25^+^FoxP3^+^ cell population in both the tumor and spleen (Figures 5a and b). However, there was no significant change in the CD4^+^CD25^+^FoxP3^+^ tumor cell population in the local pGmCSF-b7.1 treatment (Figure 5c). The combination treatment with pGmCSF-b7.1 and anti-CD25 mAb reduced both the CD4^+^CD25^+^FoxP3^+^ T-regs and the CD4^+^CD25^+^FoxP3^+^ T-cell population (activated CD4^+^ T cells; Figure 5d).

## Discussion

Malignant melanoma is one of the most aggressive forms of cancers and despite the development of molecular-targeted therapies and improvements in surgery and radiotherapy many patients succumb to recurrences and secondary's resulting in poor prognosis.^8^ The absence of an effective immune response to metastatic melanoma is thought to contribute to this poor prognosis.^10^ Several studies have described the presence and accumulation of T-regs in tumors and their increase infiltrate to be associated with this overall poor prognosis. This accumulation increases with tumor volume and is coupled with the inhibition of innate immune rejection of the tumor and precludes the proliferation of effector cells.^19, 20, 21^ Building on these observations we opted for a combinational regime of T-reg depletion and an immunotherapy treatment.

We choose an in-house plasmid encoding the cytokine GM-CSF and the costimulatory molecule human B71, which we previously demonstrated an effective recruitment of cytotoxic anti-tumor response and the permanent elimination of a poorly immunogenic fibrosarcoma tumor.^15^ B71 is usually expressed on the membrane of antigen-presenting cells, whereas tumor cells usually lack its expression and without this costimulatory signal, T cells may become clonally anergic when the T-cell receptor signal is delivered. B71-transduced tumor cells are expected to present both the antigen (T-cell receptor) and the costimulatory (CD28-mediated) signals to CD8^+^ cytotoxic T lymphocytes simultaneously, leading to efficient activation of cytotoxic T lymphocytes without requiring the assistance of CD4^+^ helper T cells.^19^ The potential of immune-based cancer treatments has been limited by negative immune-regulatory mechanisms, including tumor-derived factors that support cellular immunity and also host factors that suppress/inhibit cellular immune responses. Immune-suppressive CD4^+^CD25^+^FoxP3^+^ T-reg accumulate in primary and metastatic cancers and can prevent treatment-induced immune responses, therefore T-reg depletion represents a promising therapeutic approach. Consistent with other studies we have shown that following anti-CD25 mAb administration, CD4^+^CD25^+^FoxP3^+^ T-regs were depleted significantly (*P*<0.001) with a minimal but significant improved survival probably due to the reduction in the metastatic load.

Previously we have demonstrated an improved local response in a range of tumor types treated by electrogenetherapy with a DNA plasmid encoding for the GM-CSF and the humanB7.1 (pGmCSF-b7.1).^15, 19^ In keeping with this trend, the administration of the pGmCSF-b7.1 was associated with extended survival in the B16F10 primary tumor without being curative. This observation could potentially be due to the level of T-regs accumulating in the tumor subsequently blocking any additional immune recruitment, even with an optimal expression of GmCSF-B7.1. It is well documented that as a tumor grows the number of T-regs also accumulates. Accumulations of T-regs impinge on the effectiveness of any treatments and are associated with a negative clinical outcome. Thus, suppression of T-regs so as to allow immune recruitment to the site of the tumor post pGmCSF-B7.1 expression would be a distinct advantage to any treatment approach.

We then decided to investigate the therapeutic potential of local pGmCSF-b7.1 combined with systemic anti-CD25 mAb against established subcutaneous tumor and lung metastasis derived from the weakly immunogenic B16F10 melanoma model. It was observed that there was a modest but significant improvement of primary tumor burden and survival observed for each of the individual treatments given alone or for the combinational-treated groups. However, the combinational therapy was not effective in halting the primary tumor. The aim of all anti-cancer therapies is long-lasting responses that also prevent metastases and secondary tumors. The combination regime had a remarkable therapeutic efficacy of pre-existing lung metastases when compared to the use of either treatment alone, indicating that the suppression of T-regs with the anti-CD25 when combined with the immunotherapy pGmCSF-B7.1 allowed for an enhanced anti-tumor CD8 immune response with the decrease in the levels of metastatic nodules.

In conclusion, we have demonstrated that T-reg depletion strategy combined with an immunotherapy was effective in our model. It was observed that the immunotherapy was only effective once the T-regs were depleted. The combination of immumotherapy and T-reg depletion resulted in an effective suppression of metastasis. We conclude that the combined treatment was not effective in increasing the survival, but only effective in suppression of metastases. Although we were unable to stop the primary tumor, this treatment could fit in as a debulking therapy prior to surgery and has the potential to treat the systemic and improving prognosis of metastatic melanoma patients.
